# Clinical Profile and Factors Affecting Outcomes in Elderly Patients Admitted to the Medical Intensive Care Unit of a Tertiary Care Hospital

**DOI:** 10.7759/cureus.22136

**Published:** 2022-02-11

**Authors:** Rakesh Upparakadiyala, Subbarao Singapati, Manuj Kumar Sarkar, Swathi U

**Affiliations:** 1 General Medicine, All India Institute of Medical Sciences (AIIMS) Mangalagiri, Mangalagiri, IND; 2 Pulmonary Medicine, Sri Venkateswara (S V) Medical College, Tirupathi, Tirupathi, IND; 3 General Internal Medicine, All India Institute of Medical Sciences (AIIMS) Deoghar, Deoghar, IND; 4 Psychiatry, Pinnamaneni Siddhartha Medical College, Vijayawada, IND

**Keywords:** icu stay, disease pattern, comorbidities, medical intensive care unit, elderly patients

## Abstract

Background

In recent years, there is an increase in the proportion of the elderly population in the world. With an increase in patients' age, there is a change in the comorbidities and causes for Intensive care unit (ICU) admissions. More studies are needed to redefine healthcare delivery to elderly patients admitted to ICU.

Aims

The aims are to assess the disease pattern and outcome in elderly patients admitted to the Medical ICU and to determine factors affecting the outcomes in elderly patients admitted to the Medical ICU.

Methods

This was a retrospective cross-sectional study conducted in the Medical ICU of a tertiary care hospital for six months. Patients who met inclusion and exclusion criteria were included in this study. Data collected from medical records were statistically analysed.

Results

Out of 859 newly admitted patients to the Medical ICU, 196 (22.81%) were elderly patients (age > 60 years). The mean age of elderly patients was 69.8 ± 7.65 years. The mortality rate in elderly patients aged > 60 years was 36.70%, which was higher when compared to 23.60% in patients aged ≤ 60 years, and the correlation was statistically significant (p<0.0001). Neurological disorders (42.8%) were the most common cause of admissions, followed by renal disorders (13.26%), respiratory diseases (9.7%), and infections (9.18%). Deaths due to neurological disorders were most common (43.66%) followed by renal disorders (14.08%), infections (11.26%), and respiratory diseases (7%). The mean number of comorbidities in elderly patients was 1.99 ± 1.21. The mortality rate in elderly patients with more than three comorbidities was 56.52%, which was higher when compared to 33.52% in elderly patients with comorbidities ≤3, and the correlation was statistically insignificant (p=0.1275). The mean length of ICU stay in elderly patients was 9.14 ± 6.73 days. The length of stay in ICU was prolonged in patients with more number of comorbidities, which was statistically significant (p<0.0001). The mortality rate was higher in patients with prolonged length of stay, and the correlation was statistically significant (p=0.0013).

Conclusion

The insight over the proportion of older patients admitted to the ICU will enable policy-makers to plan accordingly. Mortality in elderly patients was high. Hence there is a need to redefine healthcare delivery to elderly patients in terms of triage and level of care in ICU. For better outcomes, risk categorisation can be done based on the number of comorbidities for optimal care. Exclusive geriatric intensive care units were needed for better care of elderly patients.

## Introduction

In recent years there is an increase in the proportion of the elderly population in the world. In India, forecasts predict the percentage of the population older than 60 years will increase from 8% in 2015 to 19% in 2050 [1]. In the past decade, there is an increase in admissions of elderly individuals into intensive care units (ICU) [2].

There is a longer prehospital delay for elderly acute myocardial infarction patients when compared to the young [3]. Age-based discrimination is seen in intensive care units. Some physicians are not ready to admit elderly patients to the Intensive care units, citing a wastage of resources. After admission, the intensity of treatment given is less in elderly patients when compared to young patients suggesting healthcare disparity [2].

With an increase in patients' age, there is a change in the comorbidities and causes for Intensive care unit admissions. Old age is recognised as a crucial independent risk factor for death [4]. Apart from old age, premorbid functional status and the severity of underlying illness also contribute to worse outcomes in this patient population [5]. Besides, prolonged hospital stay in elderly patients increases the risk of hospital-acquired infections [6].

In India, ageing of the population warrants an up-gradation of hospitals to provide optimal ICU care for elderly patients. Only a few Indian studies researched this issue. Thangam et al. conducted a study to determine the factors associated with the outcome of older patients admitted to the Geriatric ICU of a south Indian hospital [7]. Thangam et al.’s study conclude that the outcome depends on the number of comorbidities and duration of stay. More studies are needed in this area so that the data obtained from the studies can be utilised to redefine healthcare delivery in elderly patients admitted to ICU.

In view of the above context, the present study attempts to explore the disease patterns, outcomes, and factors affecting the outcome in elderly patients admitted to the Medical ICU of a large tertiary care hospital. 

Aims and objectives

The main aims and objectives are to assess the disease pattern and outcome in elderly patients admitted to the Medical ICU and to determine factors affecting the outcomes in elderly patients admitted to the Medical ICU.

## Materials and methods

Study design and population

This was a retrospective cross-sectional study conducted in the Medical ICU at Sri Venkateswara Ramnarain Ruia Government General Hospital (SVRRGGH), Tirupati, for a period of six months from September 2011 to March 2012.

Inclusion criteria

Patients aged >60 years with medical illness admitted for the first time to the Medical ICU were included in this study.

Exclusion criteria

Elderly patients with surgical/trauma-related illnesses and those who have incomplete medical records were excluded from this study.

Methodology

Patients who met the inclusion and exclusion criteria were included in this study. This study was conducted with prior permission and ethical clearance from concerned authorities. The demographic variables: age, gender, and address; and clinical variables: comorbidities, diagnosis, length of stay, and outcome were collected from medical records. Comorbidities like diabetes mellitus, hypertension, dyslipidaemia, immunocompromised status, cardiovascular disease, cerebrovascular disease, malignancy, and chronic kidney disease were noted.

Admissions, age distribution, gender distribution, the number of comorbidities, and length of stay in ICU were assessed. From the diagnosis, the primary system that was a reason for hospitalisation was identified. The outcome was measured either as discharged or deceased. Collected data were interpreted by descriptive analysis using IBM SPSS Statistical software for Windows, Version 21.0 (IBM Corp., Armonk, NY, USA). Descriptive statistics (mean±standard deviation or count/proportion) were calculated for each characteristic. The Pearson correlation test was applied to investigate relationships between the outcome and other characteristics such as age groups (61-70, 71- 80, 81-90), gender, number of comorbidities, the primary system involved, and duration of stay. A p-value of <0.05 was considered to be statistically significant.

## Results

Age and outcome

**Figure 1:**
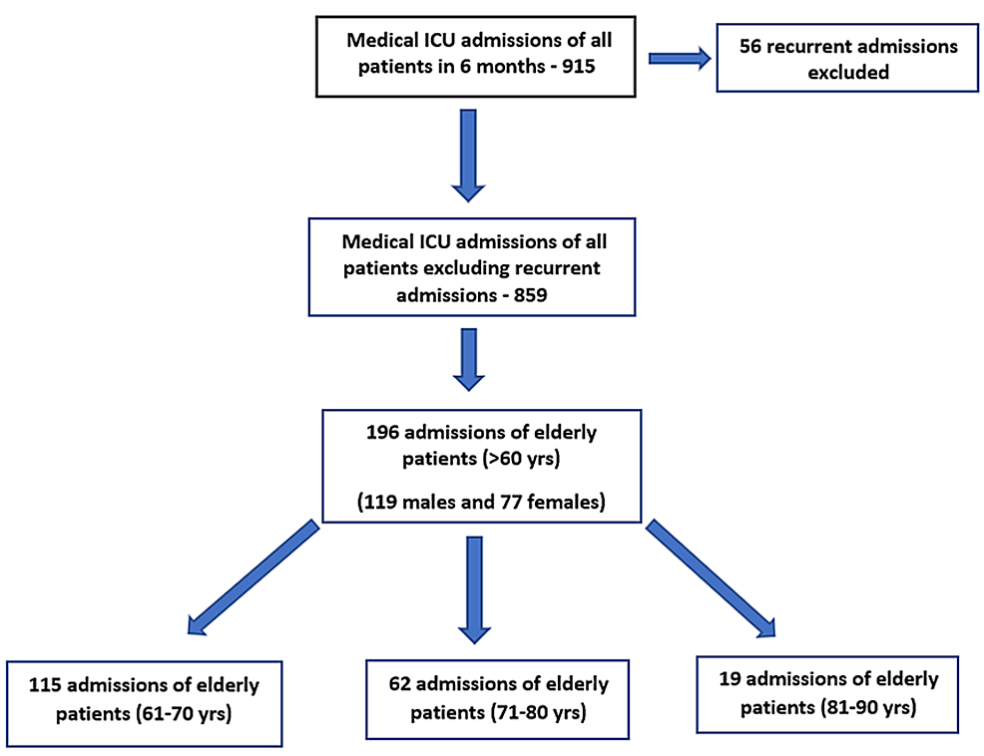
Flowchart of the study population

A total of 859 patients of all age groups were newly admitted to Medical ICU. Out of 859 patients, 196 (22.81%) were elderly patients (age > 60 years), and 663 (77.18%) patients were aged ≤60 years. Among elderly patients, 115 (58.67 %) belong to the 61-70 age group,62 (31.63%) belong to the 71-80 age group and 19 (11.73 %) belong to the 81-90 age group (Figure 1).

The mean age of elderly patients was 69.8 ± 7.65 years. The baseline characteristics of the study population were shown in Table 1.

**Table 1:** Characteristics of the study population

Characteristics	Mean±SD or Count (%)
Study population	n=196
Mean age	69.8± 7.62 years
Age group (yrs)	
61-70	115(58.67%)
71-80	62(31.63%)
81-90	19(11.73%)
Gender	
Male	119(60.71%)
Female	77(39.20%)
Primary system involved (diseases)	
Central nervous system (Cerebrovascular accidents, Meningitis and Seizures)	83(42.3%)
Respiratory system (Corpulmonale and Pneumonia)	22(9.70%)
Cardiovascular system (Heart failure)	14(7.14%)
Renal system (Acute renal failure and Chronic Kidney Disease)	26(13.26%)
Gastrointestinal tract (Hepatic encephalopathy)	5 (2.51%)
Metabolic (Diabetic Ketoacidosis, Hypoglycemia and Dyselectrolytemia)	16(8.16%)
Infections ( Cerebral malaria and Sepsis)	18(9.18%)
Haematology (Anemia)	6(3.06%)
Miscellaneous (Poisoning, Alcoholic intoxication and Snakebite)	6(3.06%)
Comorbidities	1.99± 1.21 comorbidities
Length of hospital stay	9.14 ± 6.73 days
Outcome	
Discharged	125(63.77%)
Dead	71 (36.22%)

**Figure 2:**
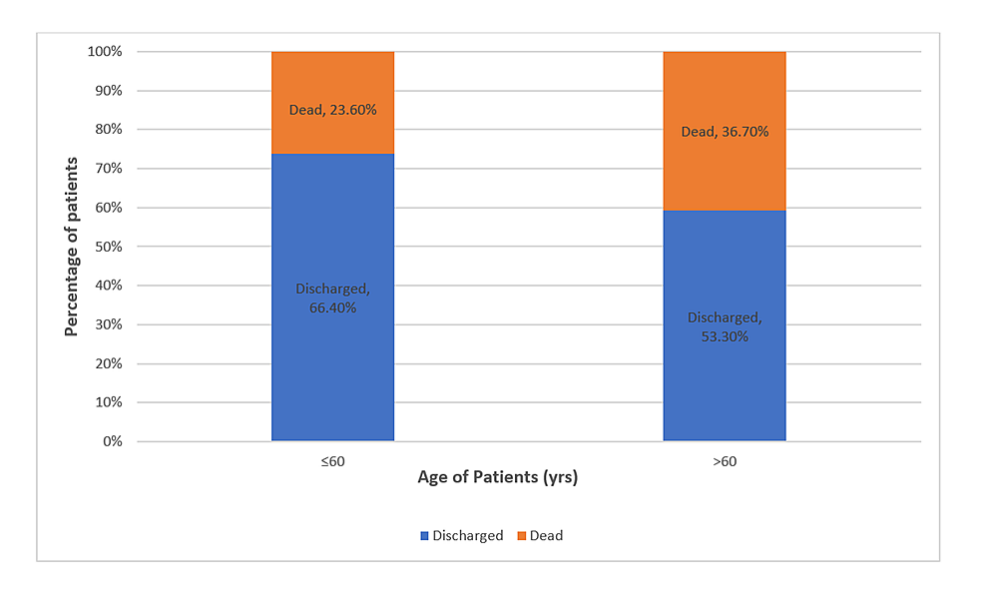
Bar graph comparing the outcome (%) in elderly patients (>60 years) and patients with age ≤60 years

The mortality rate in total admissions was 26.54% (228). The mortality rate in patients aged ≤60 years was 23.6% (157). The mortality rate in elderly patients with age > 60 years was 36.7% (71). We noticed a statistically significant association between outcomes of ≤60-year-old patients and >60-year-old patients with a p-value<0.0001 (Table 2, Figure 2).

**Table 2:** Comparison of outcomes between elderly patients (>60 years) and patients with age ≤60 years

Age group (yrs)	Outcome (n)		
	Discharged	Dead	Total
Patients age ≤60	506	157	663
Patients age>60	125	71	196
Total patients	631	228	859

 

**Figure 3:**
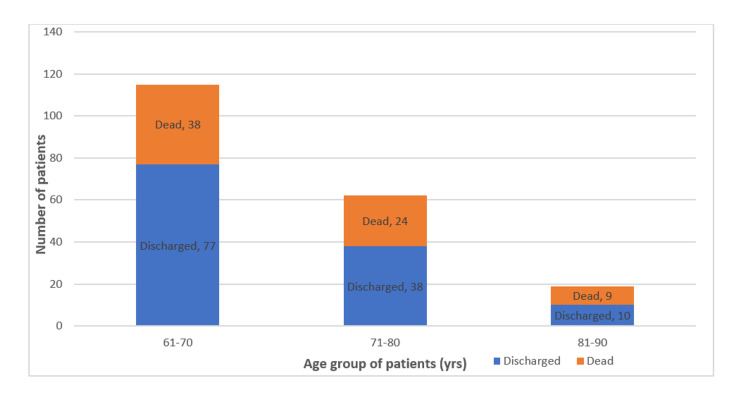
Age group-wise outcome

The mortality rate according to age groups (61-70), (71-80), and (81-90) were 33.04% (38), 38.70% (24), and 47.36% (9), respectively. There was no statistically significant correlation between age group and outcome (p=0.4293) (Figure 3).

Gender and outcome

**Figure 4:**
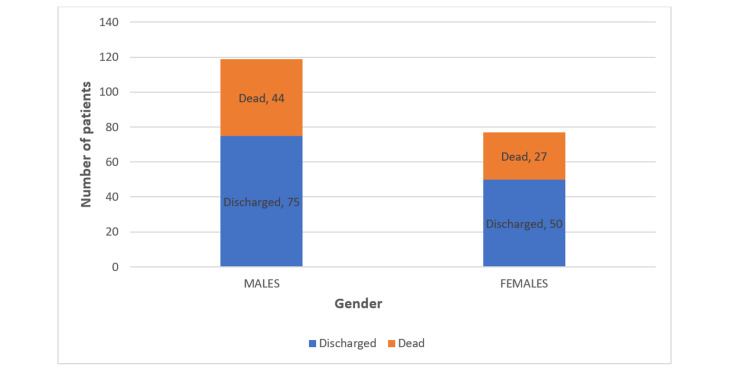
Gender and outcome

Out of 196 elderly patients, 119 (60.71%) were male, and 77 (39.20%) were female. Out of 71 deaths in elderly patients, 44 were male and 27 were female. The mortality rates among elderly male and female patients were 36.97% and 35.07%. We found no association between gender and outcome with a p-value of 0.7858, which was statistically insignificant (Figure 4).

Clinical characteristics and outcome

Eighty-three (42.8%) cases got admitted with neurological disorders. Neurological disorders were followed by renal disorders, respiratory diseases, and infections, which account for 26 (13.26%), 22 (9.70%), and 18 (9.18%) cases, respectively (Table 1).

Cerebrovascular accident (CVA) was the most common cause of admissions (27.5%) in elderly patients. Meningitis (11.73%), chronic kidney disease (9.18%), were leading causes of admissions followed by CVA.

Deaths due to neurological disorders accounted for 43.66% of total deaths. Deaths due to renal disorders, infections, and respiratory disorders were 14.08%,11.26%, and 7%, respectively. There was no statistically significant association between the primary system involved and the outcome (p=0.7255) (Table 3).

**Table 3:** Primary system involved and outcome

Primary system involved	Outcome (n)		
	Discharged (n=125)	Dead (n=71)	Total Admissions (n=196)
Nervous system	52(41.6%)	31(43.66%)	83(42.3%)
Respiratory system	17(13.6%)	5(7.04%)	22(9.70%)
Cardiovascular system	7(5.6%)	7(9.85%)	14(7.14%)
Renal system	16(12.8%)	10(14.08%)	26(13.26%)
Gastrointestinal system	3(2.4%)	2(2.81%)	5(2.51%)
Metabolic disorders	12(9.6%)	4(5.63%)	16(8.16%)
Infections	10(8%)	8(11.26%)	18(9.18%)
Hematology	5(4%)	1(1.4%)	6(3.06%)
Miscellaneous	3(2.4%)	3(4.22%)	6(3.06%)
Total	125	71	196

**Figure 5:**
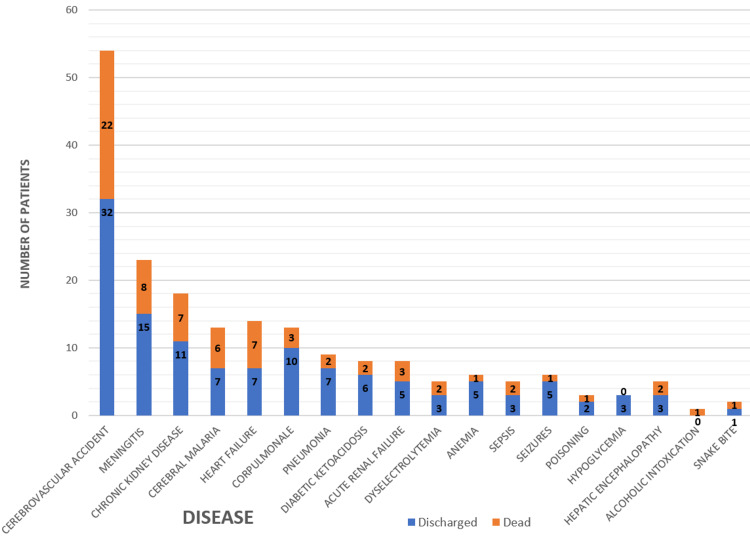
Disease-specific admissions and outcome

In elderly patients, 30.98% of deaths were due to CVAs. Meningitis (11.26%), chronic kidney disease (9.85%), heart failure (9.85%), and cerebral malaria (8.45%) were leading causes of death after CVA. There was no statistically significant correlation between the final diagnosis and outcome (p-value=0.874) (Figure 5).

Comorbidities and outcome

The mean number of comorbidities in elderly patients was 1.99 ± 1.21. The mortality rate was noted to be 56.52 % in subjects with more than 3 comorbidities and 33.52 % in subjects with 3 or less comorbidities. The association between outcome and comorbidities was statistically insignificant (p=0.1275) (Table 4).

**Table 4:** Number of comorbidities and outcome

No of comorbidities	Outcome (n)		
	Discharged	Dead	Total cases
0	19	5	24
1	33	13	46
2	37	21	58
3	26	19	45
4	9	12	21
5	1	1	2
TOTAL	125	71	196

Length of stay and comorbidities

The mean length of stay in the Medical ICU by elderly patients was 9.14 ± 6.73 days. The length of stay was prolonged in patients with three or more comorbidities when compared with patients with one or two comorbidities. The association between comorbidities and length of stay was statistically significant, with a p-value<0.0001 (Table 5).

**Table 5:** Comorbidities and length of stay in the study population

No of comorbidities	Length of stay in days				
	1-3	4-7	8-14	15-30	Total
0	13	8	1	2	24
1	11	19	14	2	46
2	8	21	18	11	58
3	9	12	16	8	45
4	2	4	6	9	21
5	0	0	0	2	2
TOTAL	43	64	55	34	196

Length of stay and outcome

When the duration of stay in ICU was correlated to the outcome, mortality rates were 28.97%, 38.18%, and 55.88% in patients who had the ICU stay of seven days, 8-14 days, and 15-30 days, respectively. The mortality rate was higher in patients with prolonged length of stay. We found a statistically significant correlation between length of stay and outcome with a p-value of 0.0013 (Table 6).

**Table 6:** Length of stay and outcome

Length of stay (days)	Outcome (n)		
	Discharged	Deaths	Total
1-3 days	24	19	43
4-7 days	52	12	64
8-14 days	34	21	55
15-30 days	15	19	34
Total	125	71	196

## Discussion

In our study, the proportion of elderly patients admitted to ICU was 22.81% of total admissions. In our study, the mean age of the elderly patients admitted to ICU was 69.8 ±7.65 years which correlated with the studies conducted by Thangam et al. (70.01± 8 years) and Lankoandé et al. (71.7± 6 years) [7,8]. The mortality rate in the study population was 36.7%, which was lower when compared to 45.1% in a study conducted by Schein et al. [9]. However, the mortality rate was 25% in a study conducted by Thangam et al., which could be attributed to different disease patterns [7]. The mortality rate in elderly patients aged > 60 years (36.7%) was much higher than in patients age≤60 years (23.6%), which was statistically significant (p<0.0001). In elderly patients, we noticed an increase in mortality with an increase in age. Similar findings were found in the study done by Yu et al. [10]. In the study by Fuchs et al., older age was regarded as a significant independent risk factor for mortality in ICU patients [4].

In our study, 60.71% of patients were male when compared to 70.5% in a study by Lankoandé et al. In the present study, The mortality rates in elderly male and female patients were 36.97% and 35.07%. In a study by Thangam et al., mortality rates in elderly males and females were 26.08% and 23.75%. In both studies, mortality among male patients was slightly higher than females, which was statistically insignificant [7]. However, a study by Fowler et al. reported a higher mortality rate in elderly females [11].

In our study, neurological disorders (42.8%), renal disorders (13.26%), respiratory diseases (9.70%), infections (9.18%), and metabolic disorders (8.16%) were common causes for admissions to medical ICU. In another study done by Lankoandé et al., neurological disorders (37.9%), sepsis (10.9%), renal disorders (6.7%), and metabolic disorders (8.4%) were common causes of admission in ICU. The disease pattern of our study is similar to the study conducted by Lankoandé et al. [8].

In our study, CVAs account for 27.50% of admissions, which is similar to 27.4% in a study conducted by Lankoandé et al. [8]. In our study, admissions in elderly patients with cardiac disease were 7.14%, which was less when compared to 24.4% in a study conducted by Thangam et al. [7]. In SVRRGG hospital where this study was conducted, Acute myocardial infarction cases were admitted to a dedicated Intensive cardiac care unit resulting in a smaller number of cardiac diseases in our study. In the present study, 43.66% of deaths were due to neurological disorders, which were high when compared to 16.3% in a study by Thangam et al. [7].

In our study, we observed an increase in mortality with an increase in the number of comorbidities, which was statistically insignificant (p=0.1275). Similar findings were present in a study by Thangam et al. [7]. 

In our study, the mean length of stay in ICU was 9.14±6.73 days, which was slightly higher when compared to 8.3±6.3 days in a study by Thangam et al. [7]. The mean length of stay was 6.6±6 days in a study by Jihane et al. [12]. The variation in the length of stay could be because of differences in disease patterns and comorbidities. In our study, we noticed an increase in mortality with an increase in the length of stay. The correlation was statistically significant (p=0.0013). Similar observations were noted in a study done by Bonfada et al. [13]. Higher mortality rates in patients with prolonged stay may be either because of severe illness or ICU-related complications. Patients with prolonged stay in ICU are more prone to develop ICU-related complications like ventilator-associated pneumonia, central venous catheter-associated bloodstream infections, urinary catheter-associated urinary tract infections, venous thromboembolism, delirium, myopathies, and neuropathies related to critical illness and stress ulcers [14].

In our study, the length of ICU stay was prolonged in patients with more number of comorbidities, which was statistically significant (p<0.0001). Similar observations were present in a study conducted by Toptas et al. [15].

The elderly are a heterogeneous population with multiple comorbidities and atypical disease presentations, which usually take a long time to diagnose. Their response to treatment is slow. Therefore, managing geriatric patients needs a multidisciplinary team and an exclusive geriatric care model [16].

The proportion of elderly patient admissions and their mortality rate in the Medical ICU were high. Hence there is a need to have exclusive geriatric intensive care units for better care.

Limitations

Our study has certain limitations. The present study included patients with medical disorders only. Patients with other conditions, such as surgical emergencies trauma-related illnesses, and critical cardiac cases were not included in this study. In this study, the post-discharge follow-up was not done.

## Conclusions

The insight over the proportion of elderly patients admitted to the ICU and their mortality rate will enable policy-makers to think of Geriatric Intensive care units. Neurological disorders (particularly CVAs) were common causes for admissions and deaths in ICU. Improvising the standards of patient care can prevent deaths due to complications of CVA, such as aspiration pneumonia. Mortality was high in patients with more comorbidities. Risk categorisation can be done based on the number of comorbidities for optimal care. There was an increase in mortality with prolonged length of stay in ICU. Length of stay can be decreased in clinically improving patients to minimise ICU-related complications. Elderly patients have multiple comorbidities and diseases with atypical presentations. Hence there is a need to redefine healthcare delivery to elderly patients in terms of triage and level of care in ICU.
